# 2,5-Bis[5-(3,5-dido­decyl­oxyphen­yl)-1,3,4-oxa­diazol-2-yl]-3,6-di­methyl­pyrazine

**DOI:** 10.1107/S2414314623000342

**Published:** 2023-01-17

**Authors:** Benedikt Joa, Dieter Schollmeyer, Heiner Detert

**Affiliations:** a University of Mainz, Department of Chemistry, Duesbergweg 10-14, 55099 Mainz, Germany; Goethe-Universität Frankfurt, Germany

**Keywords:** crystal structure, conjugated oligomer, heterocycles

## Abstract

The nearly planar mol­ecule of the title compound, C_70_H_112_N_6_O_6_, is centrosymmetric with two all-*s-trans* chains, the other two chains have an *s-cis* unit starting with the oxygen atoms. The chains are inter­digitated in the packing.

## Abstract 2

The nearly planar mol­ecule is centrosymmetric with two all-*s-trans* chains, the other two chains have an *s-cis* unit starting with the oxygen atoms. The chains are inter­digitated.

## Graphical abstract

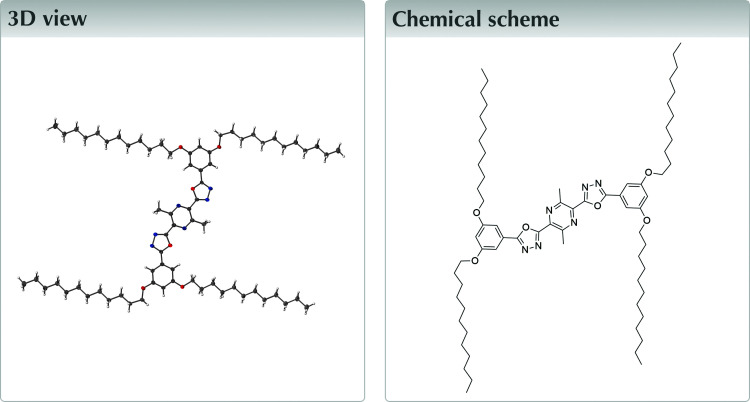

## Structure description

**Figure 1:**
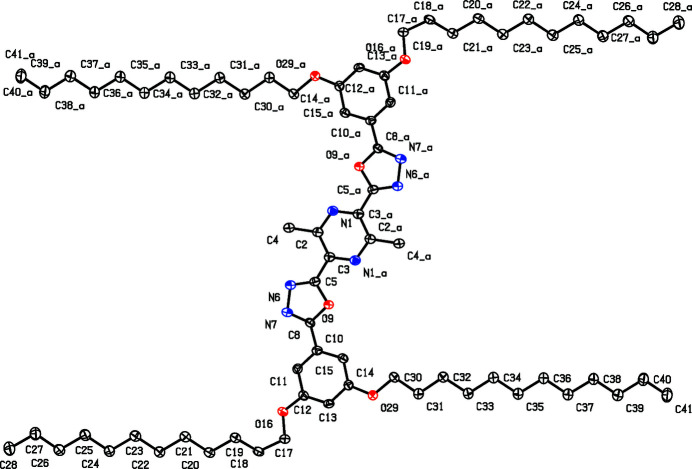
View of the title compound. Displacement ellipsoids are drawn at the 50% probability level. Atoms with suffix _a were generated by the symmetry operator −*x* + 1, −*y* + 1, −*z* + 1. H atoms were omitted for clarity.

**Figure 2:**
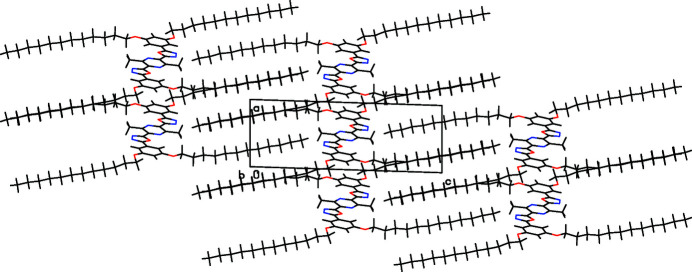
Partial packing diagram. View along the *b*-axis.

Electron-deficient conjugated oligomers are inter­esting as electron-transporting mat­erials in organic electronics (Müllen & Wegner, 1998 ▸). The combination of rigid segments with flexible side chains may cause mesomorphism (Vorländer, 1923 ▸). Alternating aryl­ene-1,3,4-oxa­diazo­lylene oligomers were first prepared by a Huisgen reaction (Sauer *et al.*, 1960 ▸). The title compound (Fig. 1 ▸) was prepared *via* twofold acyl­ation of the di­alk­oxy­phenyl-tetra­zole with a pyrazinedi­carb­oxy­lic acid dichloride and thermal ring transformation. The centrosymmetric mol­ecule is located parallel to the (110) plane, the five aromatic rings are almost coplanar with torsion angles of −0.4 (4)° (N6—C5—C3—C2) and −2.0 (4)° (C11—C10—C8—N7). Two dodec­yloxy chains per mol­ecule are completely all-*s-trans* organized whereas the other pair shows an *s-cis*-conformation of the O16—C17—C18—C19 unit. Torsion angles at the all-*trans* chains are 179.3 (2) for C13—C14—O29—C30, 177.9 (5)° for C14—O29—C30—C31 and 178.1 (2)° for O29—C30—C31—C32 but for the other chain, 15.8 (3)° (C17—O16—C12—C13), 176.2 (2)° (C12—O16—C17—C18), and −55.3 (3)° for the O16—C17—C18—C19 unit. The packing is controlled by inter­action of the aliphatic chains (Fig. 2 ▸), which are arranged in two planes subtending a dihedral angle of 7.63 (19)°. Deviation from planarity within the chains is only −0.481 (2) Å at C30 but −1.84 (2) Å at C18. Alternating all-*trans* chains and those with the *s-cis* unit are inter­digitated. The aromatic units of mol­ecules in different planes are displaced, C13 (phen­yl) is located above the pyrazine centroid.

## Synthesis and crystallization

The title compound was prepared *via* a Huisgen reaction (Sauer *et al.*, 1960 ▸) of a central di­carb­oxy­lic acid chloride and tetra­zoles. 3,6-Di­methyl­pyrazine-2,5-di­carb­oxy­lic acid (0.1 g, 0.51 mmol) (Brunner *et al.*, 1998 ▸) was dissolved in thionyl chloride (5 ml) and after refluxing for 2 h, the reagent was evaporated, residues were removed by codistillation with toluene (10 ml). 5-(3,5-Dido­decyl­oxyphen­yl)tetra­zole (0.52 g, 1.02 mmol) (Rieth *et al.*, 2015 ▸) and pyridine (3 ml) were added and the mixture refluxed for 24 h. Thereafter, water and diluted hydro­chloric acid were added and the product isolated *via* extraction with chloro­form, drying with MgSO_4_ and chromatography on silica using ethyl acetate as an eluent. Yield: 20 mg, 3% of a colorless material with m.p. = 399 K. IR (ATR): 2916, 2848, 1602, 1544, 1468, 1442, 1429, 1174; ^1^H NMR (CDCl_3_, 400 MHz; δ = 7.34 (*d*, *J* = 2.2 Hz, 4 H), 6.66 (*t*, *J* = 2.3 Hz, 2 H), 4.04 (*t*, *J* = 6.5 Hz, 8 H), 3.17 (*s*, 6 H), 1.82 (*qui*, *J* = 6.9 Hz, 8 H), 1.55-1.42 (*qui*,≃ 8 H), 1.42-1.20 (*qui*, ≃ 64 H), 0.93-0.83 (*t*, ≃ 12 H). Whereas the absorption spectrum of the title compound in cyclo­hexane peaks at 348 nm, three maxima appear in methanol (346, 361, and 379 nm), indicating aggregation. The emission of a solution in cyclo­hexane is centered at 403 nm, and in toluene at 409 nm. Crystallization was *via* slow evaporation of a solution in chloro­form/2-propanol.

## Refinement

Crystal data, data collection and structure refinement details are summarized in Table 1 ▸.

## Supplementary Material

Crystal structure: contains datablock(s) I, global. DOI: 10.1107/S2414314623000342/bt4131sup1.cif

Structure factors: contains datablock(s) I. DOI: 10.1107/S2414314623000342/bt4131Isup2.hkl

CCDC reference: 2235881

Additional supporting information:  crystallographic information; 3D view; checkCIF report

## Figures and Tables

**Table 1:** Experimental details

Crystal data	
Chemical formula	C_70_H_112_N_6_O_6_
*M*_r_	1133.65
Crystal system, space group	Triclinic, *P* 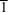
Temperature (K)	120
*a*, *b*, *c* (Å)	8.1119 (3), 9.5566 (3), 22.2332 (8)
α, β, γ (°)	90.553 (3), 91.592 (3), 106.047 (3)
*V* (Å^3^)	1655.50 (10)
*Z*	1
Radiation type	Cu *K*α
μ (mm^−1^)	0.56
Crystal size (mm)	0.14 × 0.08 × 0.03

Data collection	
Diffractometer	Stoe Stadivari
No. of measured, independent and observed [*I* > 2σ(*I*)] reflections	46250, 5582, 3360
*R*_int_	0.090
(sin θ/λ)_max_ (Å^−1^)	0.601

Refinement	
*R*[*F* ^2^ > 2σ(*F* ^2^)], *wR*(*F* ^2^), *S*	0.050, 0.143, 0.94
No. of reflections	5582
No. of parameters	373
H-atom treatment	H-atom parameters constrained
Δρ_max_, Δρ_min_ (e Å^−3^)	0.57, −0.21

Computer programs: *X-AREA* *WinXpose*, *Recipe* and*Integrate* (Stoe & Cie, 2019 ▸), *SHELXT2014* (Sheldrick, 2015*a* ▸), *SHELXL2018/3* (Sheldrick, 2015*b* ▸) and *PLATON* (Spek, 2020 ▸).

## full crystallographic data

### Crystal data

**Table:**

C_70_H_112_N_6_O_6_	*F*(000) = 622
*M**_r_* = 1133.65	*D*_x_ = 1.137 Mg m^−^^3^
Triclinic, *P*1	Melting point: 399 K
*a* = 8.1119 (3) Å	Cu *K*α radiation, λ = 1.54178 Å
*b* = 9.5566 (3) Å	Cell parameters from 12421 reflections
*c* = 22.2332 (8) Å	θ = 2.0–71.5°
α = 90.553 (3)°	µ = 0.56 mm^−^^1^
β = 91.592 (3)°	*T* = 120 K
γ = 106.047 (3)°	Block, colorless
*V* = 1655.50 (10) Å^3^	0.14 × 0.08 × 0.03 mm
*Z* = 1	

### Data collection

**Table:**

Stoe Stadivari diffractometer	3360 reflections with *I* > 2σ(*I*)
Radiation source: microfocus tube	*R*_int_ = 0.090
Detector resolution: 13.33 pixels mm^-1^	θ_max_ = 68.0°, θ_min_ = 2.0°
rotation method, ω scans	*h* = −9→9
46250 measured reflections	*k* = −11→11
5582 independent reflections	*l* = −26→26

### Refinement

**Table:**

Refinement on *F*^2^	Primary atom site location: dual
Least-squares matrix: full	Hydrogen site location: inferred from neighbouring sites
*R*[*F*^2^ > 2σ(*F*^2^)] = 0.050	H-atom parameters constrained
*wR*(*F*^2^) = 0.143	*w* = 1/[σ^2^(*F*_o_^2^) + (0.0817*P*)^2^] where *P* = (*F*_o_^2^ + 2*F*_c_^2^)/3
*S* = 0.94	(Δ/σ)_max_ < 0.001
5582 reflections	Δρ_max_ = 0.57 e Å^−^^3^
373 parameters	Δρ_min_ = −0.21 e Å^−^^3^
0 restraints	

### Special details

**Table:**

Geometry. All esds (except the esd in the dihedral angle between two l.s. planes) are estimated using the full covariance matrix. The cell esds are taken into account individually in the estimation of esds in distances, angles and torsion angles; correlations between esds in cell parameters are only used when they are defined by crystal symmetry. An approximate (isotropic) treatment of cell esds is used for estimating esds involving l.s. planes.
Refinement. Hydrogen atoms were placed at calculated positions and were refined in the riding-model approximation with C_aromatic_–H = 0.95 Å, C_methylene_–H = 0.99 Å, and C_methyl_–H = 0.98 Å and with *U*_iso_(H) = 1.2 *U*_eq_(C) or *U*_iso_(H) = 1.5 *U*_eq_(C_methyl_). The methyl groups were allowed to rotate but not to tip.

### Fractional atomic coordinates and isotropic or equivalent isotropic displacement parameters (Å^2^)

**Table:**

	*x*	*y*	*z*	*U*_iso_*/*U*_eq_	
N1	0.4330 (2)	0.5972 (2)	0.53110 (8)	0.0250 (4)	
C2	0.4840 (3)	0.4951 (2)	0.56115 (10)	0.0248 (5)	
C3	0.5511 (3)	0.3976 (2)	0.52863 (10)	0.0240 (5)	
C4	0.4685 (3)	0.4954 (2)	0.62820 (10)	0.0289 (5)	
H4A	0.417809	0.572892	0.640336	0.043*	
H4B	0.582537	0.512466	0.647519	0.043*	
H4C	0.394915	0.401114	0.640563	0.043*	
C5	0.6013 (3)	0.2776 (2)	0.55730 (10)	0.0244 (5)	
N6	0.5959 (3)	0.2403 (2)	0.61295 (8)	0.0300 (5)	
N7	0.6494 (3)	0.1122 (2)	0.61502 (8)	0.0304 (5)	
C8	0.6824 (3)	0.0848 (2)	0.56041 (9)	0.0245 (5)	
O9	0.6569 (2)	0.18470 (16)	0.52090 (6)	0.0244 (4)	
C10	0.7459 (3)	−0.0346 (2)	0.53796 (10)	0.0236 (5)	
C11	0.7808 (3)	−0.1302 (2)	0.57946 (10)	0.0247 (5)	
H11	0.757950	−0.120829	0.620758	0.030*	
C12	0.8500 (3)	−0.2404 (2)	0.56000 (9)	0.0241 (5)	
C13	0.8827 (3)	−0.2539 (2)	0.49996 (9)	0.0240 (5)	
H13	0.931369	−0.328264	0.486925	0.029*	
C14	0.8443 (3)	−0.1580 (2)	0.45843 (9)	0.0228 (5)	
C15	0.7753 (3)	−0.0471 (2)	0.47684 (10)	0.0240 (5)	
H15	0.749062	0.018229	0.448630	0.029*	
O16	0.8803 (2)	−0.32816 (17)	0.60445 (6)	0.0287 (4)	
C17	0.9903 (3)	−0.4195 (2)	0.59294 (10)	0.0277 (5)	
H17A	1.103735	−0.360190	0.580051	0.033*	
H17B	0.938976	−0.492003	0.560728	0.033*	
C18	1.0092 (3)	−0.4953 (2)	0.65108 (10)	0.0291 (5)	
H18A	1.089988	−0.554965	0.645231	0.035*	
H18B	0.896548	−0.562135	0.660717	0.035*	
C19	1.0742 (3)	−0.3907 (3)	0.70426 (10)	0.0304 (5)	
H19A	0.986994	−0.339312	0.713321	0.036*	
H19B	1.180210	−0.316845	0.693067	0.036*	
C20	1.1118 (3)	−0.4684 (3)	0.76074 (10)	0.0307 (6)	
H20A	1.214195	−0.503073	0.754146	0.037*	
H20B	1.013675	−0.554834	0.766994	0.037*	
C21	1.1432 (3)	−0.3723 (3)	0.81777 (10)	0.0333 (6)	
H21A	1.240036	−0.284909	0.811395	0.040*	
H21B	1.039951	−0.339102	0.824945	0.040*	
C22	1.1837 (3)	−0.4506 (3)	0.87345 (10)	0.0323 (6)	
H22A	1.289125	−0.480808	0.866759	0.039*	
H22B	1.088599	−0.539766	0.878963	0.039*	
C23	1.2092 (3)	−0.3569 (3)	0.93082 (10)	0.0334 (6)	
H23A	1.104016	−0.326191	0.937327	0.040*	
H23B	1.304701	−0.267990	0.925339	0.040*	
C24	1.2490 (3)	−0.4354 (3)	0.98696 (10)	0.0319 (6)	
H24A	1.153882	−0.524675	0.992303	0.038*	
H24B	1.354663	−0.465467	0.980597	0.038*	
C25	1.2732 (3)	−0.3418 (3)	1.04433 (10)	0.0335 (6)	
H25A	1.367141	−0.251867	1.038704	0.040*	
H25B	1.166897	−0.312784	1.050905	0.040*	
C26	1.3151 (3)	−0.4182 (3)	1.10030 (10)	0.0338 (6)	
H26A	1.221315	−0.508143	1.106018	0.041*	
H26B	1.421681	−0.446844	1.093908	0.041*	
C27	1.3385 (4)	−0.3235 (3)	1.15719 (10)	0.0374 (6)	
H27A	1.231819	−0.294916	1.163560	0.045*	
H27B	1.432107	−0.233484	1.151410	0.045*	
C28	1.3808 (4)	−0.3996 (3)	1.21353 (11)	0.0446 (7)	
H28A	1.287662	−0.488016	1.220028	0.067*	
H28B	1.393540	−0.333864	1.248507	0.067*	
H28C	1.488134	−0.425691	1.208107	0.067*	
O29	0.8808 (2)	−0.18117 (16)	0.40032 (6)	0.0284 (4)	
C30	0.8427 (3)	−0.0874 (2)	0.35478 (9)	0.0287 (5)	
H30A	0.910389	0.014655	0.362777	0.034*	
H30B	0.719233	−0.092015	0.354322	0.034*	
C31	0.8895 (3)	−0.1410 (3)	0.29551 (10)	0.0326 (6)	
H31A	0.825290	−0.244774	0.289450	0.039*	
H31B	1.013475	−0.134541	0.296749	0.039*	
C32	0.8501 (4)	−0.0542 (3)	0.24239 (10)	0.0329 (6)	
H32A	0.734167	−0.040872	0.246459	0.039*	
H32B	0.933784	0.043378	0.243139	0.039*	
C33	0.8580 (3)	−0.1298 (3)	0.18232 (10)	0.0320 (6)	
H33A	0.776254	−0.228237	0.182322	0.038*	
H33B	0.974638	−0.141789	0.178384	0.038*	
C34	0.8164 (3)	−0.0486 (3)	0.12775 (10)	0.0319 (6)	
H34A	0.908077	0.043686	0.123919	0.038*	
H34B	0.707375	−0.023895	0.134115	0.038*	
C35	0.8002 (3)	−0.1378 (3)	0.06942 (9)	0.0317 (6)	
H35A	0.709602	−0.230584	0.073611	0.038*	
H35B	0.909714	−0.161648	0.063027	0.038*	
C36	0.7566 (3)	−0.0591 (3)	0.01426 (9)	0.0321 (6)	
H36A	0.648880	−0.032642	0.021052	0.039*	
H36B	0.849030	0.032104	0.009195	0.039*	
C37	0.7354 (3)	−0.1514 (3)	−0.04338 (10)	0.0332 (6)	
H37A	0.843351	−0.177654	−0.050164	0.040*	
H37B	0.643403	−0.242829	−0.038181	0.040*	
C38	0.6913 (3)	−0.0740 (3)	−0.09865 (10)	0.0324 (6)	
H38A	0.784213	0.016683	−0.104200	0.039*	
H38B	0.584358	−0.046434	−0.091618	0.039*	
C39	0.6675 (4)	−0.1669 (3)	−0.15610 (10)	0.0349 (6)	
H39A	0.576900	−0.258829	−0.150162	0.042*	
H39B	0.775603	−0.192197	−0.163757	0.042*	
C40	0.6186 (4)	−0.0908 (3)	−0.21120 (10)	0.0377 (6)	
H40A	0.513767	−0.060992	−0.202886	0.045*	
H40B	0.712046	−0.001719	−0.218608	0.045*	
C41	0.5864 (4)	−0.1877 (3)	−0.26715 (11)	0.0446 (7)	
H41A	0.691687	−0.213281	−0.276828	0.054*	
H41B	0.551884	−0.135694	−0.300897	0.054*	
H41C	0.494847	−0.276608	−0.259900	0.054*	

### Atomic displacement parameters (Å^2^)

**Table:**

	*U*^11^	*U*^22^	*U*^33^	*U*^12^	*U*^13^	*U*^23^
N1	0.0246 (11)	0.0213 (10)	0.0288 (11)	0.0063 (10)	−0.0022 (8)	−0.0021 (7)
C2	0.0223 (13)	0.0218 (12)	0.0303 (12)	0.0063 (12)	−0.0024 (9)	−0.0013 (9)
C3	0.0206 (12)	0.0198 (12)	0.0301 (12)	0.0037 (11)	−0.0020 (9)	−0.0028 (9)
C4	0.0338 (14)	0.0253 (12)	0.0302 (13)	0.0125 (12)	0.0005 (10)	−0.0015 (9)
C5	0.0226 (12)	0.0220 (12)	0.0294 (12)	0.0078 (11)	−0.0016 (9)	−0.0044 (9)
N6	0.0388 (13)	0.0280 (11)	0.0280 (11)	0.0177 (11)	−0.0011 (9)	−0.0018 (8)
N7	0.0387 (13)	0.0265 (11)	0.0310 (11)	0.0174 (11)	0.0000 (9)	0.0016 (8)
C8	0.0282 (13)	0.0211 (12)	0.0253 (12)	0.0088 (12)	−0.0022 (9)	0.0027 (8)
O9	0.0286 (9)	0.0228 (8)	0.0245 (8)	0.0118 (8)	−0.0002 (6)	−0.0004 (6)
C10	0.0209 (12)	0.0223 (12)	0.0283 (12)	0.0076 (11)	−0.0012 (9)	−0.0022 (9)
C11	0.0253 (13)	0.0248 (12)	0.0237 (11)	0.0066 (12)	−0.0006 (9)	−0.0025 (8)
C12	0.0232 (13)	0.0227 (12)	0.0262 (12)	0.0060 (12)	−0.0029 (9)	0.0017 (9)
C13	0.0255 (13)	0.0190 (11)	0.0284 (12)	0.0080 (11)	−0.0009 (9)	−0.0025 (8)
C14	0.0234 (12)	0.0206 (12)	0.0227 (11)	0.0034 (11)	−0.0020 (8)	−0.0007 (8)
C15	0.0251 (13)	0.0201 (11)	0.0267 (11)	0.0067 (11)	−0.0032 (9)	0.0016 (8)
O16	0.0358 (10)	0.0304 (9)	0.0262 (8)	0.0192 (9)	0.0016 (7)	0.0043 (6)
C17	0.0313 (14)	0.0284 (13)	0.0277 (12)	0.0157 (12)	0.0000 (9)	−0.0006 (9)
C18	0.0315 (14)	0.0265 (12)	0.0325 (13)	0.0141 (12)	−0.0036 (10)	0.0026 (9)
C19	0.0366 (15)	0.0282 (13)	0.0290 (12)	0.0138 (13)	−0.0020 (10)	0.0016 (9)
C20	0.0360 (15)	0.0279 (13)	0.0311 (13)	0.0137 (13)	−0.0025 (10)	0.0020 (9)
C21	0.0402 (16)	0.0334 (14)	0.0299 (13)	0.0164 (14)	−0.0028 (10)	0.0006 (10)
C22	0.0392 (15)	0.0302 (13)	0.0291 (13)	0.0127 (13)	−0.0018 (10)	0.0006 (9)
C23	0.0391 (16)	0.0309 (14)	0.0318 (13)	0.0127 (14)	−0.0018 (11)	0.0012 (10)
C24	0.0382 (15)	0.0303 (14)	0.0279 (13)	0.0113 (13)	−0.0023 (10)	0.0005 (10)
C25	0.0386 (16)	0.0320 (14)	0.0303 (13)	0.0110 (14)	−0.0032 (10)	0.0006 (10)
C26	0.0367 (15)	0.0336 (14)	0.0323 (13)	0.0118 (13)	−0.0020 (11)	0.0024 (10)
C27	0.0414 (16)	0.0399 (15)	0.0299 (13)	0.0101 (15)	−0.0022 (11)	0.0006 (10)
C28	0.0494 (18)	0.0547 (18)	0.0323 (14)	0.0194 (16)	−0.0047 (12)	0.0015 (12)
O29	0.0394 (10)	0.0268 (9)	0.0226 (8)	0.0150 (9)	0.0000 (7)	0.0007 (6)
C30	0.0353 (14)	0.0278 (13)	0.0252 (12)	0.0127 (13)	−0.0026 (10)	0.0026 (9)
C31	0.0432 (16)	0.0324 (14)	0.0250 (12)	0.0151 (14)	0.0007 (10)	0.0007 (9)
C32	0.0447 (16)	0.0303 (13)	0.0267 (12)	0.0156 (13)	0.0006 (10)	0.0014 (9)
C33	0.0407 (16)	0.0318 (13)	0.0250 (12)	0.0130 (13)	−0.0006 (10)	0.0016 (9)
C34	0.0379 (15)	0.0336 (14)	0.0261 (12)	0.0132 (13)	0.0005 (10)	0.0013 (9)
C35	0.0371 (15)	0.0328 (14)	0.0261 (13)	0.0112 (13)	−0.0008 (10)	0.0001 (10)
C36	0.0382 (15)	0.0362 (14)	0.0241 (12)	0.0142 (14)	−0.0004 (10)	0.0004 (10)
C37	0.0393 (15)	0.0349 (14)	0.0268 (12)	0.0129 (14)	−0.0026 (10)	−0.0015 (10)
C38	0.0384 (15)	0.0344 (14)	0.0250 (12)	0.0115 (13)	−0.0024 (10)	−0.0008 (9)
C39	0.0401 (16)	0.0358 (14)	0.0294 (13)	0.0117 (14)	−0.0008 (11)	−0.0013 (10)
C40	0.0452 (17)	0.0406 (15)	0.0269 (13)	0.0115 (15)	−0.0023 (11)	−0.0022 (10)
C41	0.0508 (19)	0.0566 (18)	0.0275 (14)	0.0169 (17)	−0.0020 (12)	−0.0019 (12)

### Geometric parameters (Å, º)

**Table:**

N1—C2	1.338 (3)	C25—C26	1.526 (3)
N1—C3^i^	1.338 (3)	C25—H25A	0.9900
C2—C3	1.406 (3)	C25—H25B	0.9900
C2—C4	1.499 (3)	C26—C27	1.524 (3)
C3—C5	1.465 (3)	C26—H26A	0.9900
C4—H4A	0.9800	C26—H26B	0.9900
C4—H4B	0.9800	C27—C28	1.532 (3)
C4—H4C	0.9800	C27—H27A	0.9900
C5—N6	1.290 (3)	C27—H27B	0.9900
C5—O9	1.369 (2)	C28—H28A	0.9800
N6—N7	1.408 (2)	C28—H28B	0.9800
N7—C8	1.290 (3)	C28—H28C	0.9800
C8—O9	1.357 (2)	O29—C30	1.441 (2)
C8—C10	1.465 (3)	C30—C31	1.505 (3)
C10—C11	1.382 (3)	C30—H30A	0.9900
C10—C15	1.396 (3)	C30—H30B	0.9900
C11—C12	1.394 (3)	C31—C32	1.526 (3)
C11—H11	0.9500	C31—H31A	0.9900
C12—O16	1.362 (3)	C31—H31B	0.9900
C12—C13	1.380 (3)	C32—C33	1.523 (3)
C13—C14	1.395 (3)	C32—H32A	0.9900
C13—H13	0.9500	C32—H32B	0.9900
C14—O29	1.363 (3)	C33—C34	1.526 (3)
C14—C15	1.391 (3)	C33—H33A	0.9900
C15—H15	0.9500	C33—H33B	0.9900
O16—C17	1.437 (2)	C34—C35	1.527 (3)
C17—C18	1.513 (3)	C34—H34A	0.9900
C17—H17A	0.9900	C34—H34B	0.9900
C17—H17B	0.9900	C35—C36	1.528 (3)
C18—C19	1.525 (3)	C35—H35A	0.9900
C18—H18A	0.9900	C35—H35B	0.9900
C18—H18B	0.9900	C36—C37	1.527 (3)
C19—C20	1.530 (3)	C36—H36A	0.9900
C19—H19A	0.9900	C36—H36B	0.9900
C19—H19B	0.9900	C37—C38	1.525 (3)
C20—C21	1.532 (3)	C37—H37A	0.9900
C20—H20A	0.9900	C37—H37B	0.9900
C20—H20B	0.9900	C38—C39	1.525 (3)
C21—C22	1.527 (3)	C38—H38A	0.9900
C21—H21A	0.9900	C38—H38B	0.9900
C21—H21B	0.9900	C39—C40	1.529 (3)
C22—C23	1.527 (3)	C39—H39A	0.9900
C22—H22A	0.9900	C39—H39B	0.9900
C22—H22B	0.9900	C40—C41	1.516 (3)
C23—C24	1.534 (3)	C40—H40A	0.9900
C23—H23A	0.9900	C40—H40B	0.9900
C23—H23B	0.9900	C41—H41A	0.9800
C24—C25	1.527 (3)	C41—H41B	0.9800
C24—H24A	0.9900	C41—H41C	0.9800
C24—H24B	0.9900		
			
C2—N1—C3^i^	118.49 (19)	C24—C25—H25B	108.8
N1—C2—C3	118.7 (2)	H25A—C25—H25B	107.7
N1—C2—C4	116.83 (18)	C27—C26—C25	113.1 (2)
C3—C2—C4	124.49 (19)	C27—C26—H26A	109.0
N1^i^—C3—C2	122.8 (2)	C25—C26—H26A	109.0
N1^i^—C3—C5	114.69 (19)	C27—C26—H26B	109.0
C2—C3—C5	122.4 (2)	C25—C26—H26B	109.0
C2—C4—H4A	109.5	H26A—C26—H26B	107.8
C2—C4—H4B	109.5	C26—C27—C28	113.3 (2)
H4A—C4—H4B	109.5	C26—C27—H27A	108.9
C2—C4—H4C	109.5	C28—C27—H27A	108.9
H4A—C4—H4C	109.5	C26—C27—H27B	108.9
H4B—C4—H4C	109.5	C28—C27—H27B	108.9
N6—C5—O9	112.67 (18)	H27A—C27—H27B	107.7
N6—C5—C3	129.76 (19)	C27—C28—H28A	109.5
O9—C5—C3	117.52 (18)	C27—C28—H28B	109.5
C5—N6—N7	106.03 (17)	H28A—C28—H28B	109.5
C8—N7—N6	106.06 (17)	C27—C28—H28C	109.5
N7—C8—O9	113.02 (18)	H28A—C28—H28C	109.5
N7—C8—C10	128.27 (19)	H28B—C28—H28C	109.5
O9—C8—C10	118.69 (18)	C14—O29—C30	118.34 (16)
C8—O9—C5	102.21 (16)	O29—C30—C31	106.77 (17)
C11—C10—C15	121.71 (19)	O29—C30—H30A	110.4
C11—C10—C8	117.78 (19)	C31—C30—H30A	110.4
C15—C10—C8	120.48 (19)	O29—C30—H30B	110.4
C10—C11—C12	119.2 (2)	C31—C30—H30B	110.4
C10—C11—H11	120.4	H30A—C30—H30B	108.6
C12—C11—H11	120.4	C30—C31—C32	112.61 (19)
O16—C12—C13	125.20 (19)	C30—C31—H31A	109.1
O16—C12—C11	114.55 (19)	C32—C31—H31A	109.1
C13—C12—C11	120.25 (19)	C30—C31—H31B	109.1
C12—C13—C14	119.89 (19)	C32—C31—H31B	109.1
C12—C13—H13	120.1	H31A—C31—H31B	107.8
C14—C13—H13	120.1	C33—C32—C31	112.02 (19)
O29—C14—C15	123.95 (19)	C33—C32—H32A	109.2
O29—C14—C13	115.19 (18)	C31—C32—H32A	109.2
C15—C14—C13	120.8 (2)	C33—C32—H32B	109.2
C14—C15—C10	118.08 (19)	C31—C32—H32B	109.2
C14—C15—H15	121.0	H32A—C32—H32B	107.9
C10—C15—H15	121.0	C32—C33—C34	114.08 (19)
C12—O16—C17	118.96 (17)	C32—C33—H33A	108.7
O16—C17—C18	106.94 (17)	C34—C33—H33A	108.7
O16—C17—H17A	110.3	C32—C33—H33B	108.7
C18—C17—H17A	110.3	C34—C33—H33B	108.7
O16—C17—H17B	110.3	H33A—C33—H33B	107.6
C18—C17—H17B	110.3	C33—C34—C35	112.41 (19)
H17A—C17—H17B	108.6	C33—C34—H34A	109.1
C17—C18—C19	113.50 (19)	C35—C34—H34A	109.1
C17—C18—H18A	108.9	C33—C34—H34B	109.1
C19—C18—H18A	108.9	C35—C34—H34B	109.1
C17—C18—H18B	108.9	H34A—C34—H34B	107.9
C19—C18—H18B	108.9	C34—C35—C36	113.29 (19)
H18A—C18—H18B	107.7	C34—C35—H35A	108.9
C18—C19—C20	112.56 (19)	C36—C35—H35A	108.9
C18—C19—H19A	109.1	C34—C35—H35B	108.9
C20—C19—H19A	109.1	C36—C35—H35B	108.9
C18—C19—H19B	109.1	H35A—C35—H35B	107.7
C20—C19—H19B	109.1	C37—C36—C35	112.69 (19)
H19A—C19—H19B	107.8	C37—C36—H36A	109.1
C19—C20—C21	113.68 (19)	C35—C36—H36A	109.1
C19—C20—H20A	108.8	C37—C36—H36B	109.1
C21—C20—H20A	108.8	C35—C36—H36B	109.1
C19—C20—H20B	108.8	H36A—C36—H36B	107.8
C21—C20—H20B	108.8	C38—C37—C36	113.07 (19)
H20A—C20—H20B	107.7	C38—C37—H37A	109.0
C22—C21—C20	112.97 (19)	C36—C37—H37A	109.0
C22—C21—H21A	109.0	C38—C37—H37B	109.0
C20—C21—H21A	109.0	C36—C37—H37B	109.0
C22—C21—H21B	109.0	H37A—C37—H37B	107.8
C20—C21—H21B	109.0	C37—C38—C39	113.1 (2)
H21A—C21—H21B	107.8	C37—C38—H38A	109.0
C21—C22—C23	113.19 (19)	C39—C38—H38A	109.0
C21—C22—H22A	108.9	C37—C38—H38B	109.0
C23—C22—H22A	108.9	C39—C38—H38B	109.0
C21—C22—H22B	108.9	H38A—C38—H38B	107.8
C23—C22—H22B	108.9	C38—C39—C40	113.1 (2)
H22A—C22—H22B	107.8	C38—C39—H39A	109.0
C22—C23—C24	113.40 (19)	C40—C39—H39A	109.0
C22—C23—H23A	108.9	C38—C39—H39B	109.0
C24—C23—H23A	108.9	C40—C39—H39B	109.0
C22—C23—H23B	108.9	H39A—C39—H39B	107.8
C24—C23—H23B	108.9	C41—C40—C39	112.3 (2)
H23A—C23—H23B	107.7	C41—C40—H40A	109.1
C25—C24—C23	113.27 (19)	C39—C40—H40A	109.1
C25—C24—H24A	108.9	C41—C40—H40B	109.1
C23—C24—H24A	108.9	C39—C40—H40B	109.1
C25—C24—H24B	108.9	H40A—C40—H40B	107.9
C23—C24—H24B	108.9	C40—C41—H41A	109.5
H24A—C24—H24B	107.7	C40—C41—H41B	109.5
C26—C25—C24	113.68 (19)	H41A—C41—H41B	109.5
C26—C25—H25A	108.8	C40—C41—H41C	109.5
C24—C25—H25A	108.8	H41A—C41—H41C	109.5
C26—C25—H25B	108.8	H41B—C41—H41C	109.5
			
C3^i^—N1—C2—C3	−0.3 (4)	O29—C14—C15—C10	−179.3 (2)
C3^i^—N1—C2—C4	178.4 (2)	C13—C14—C15—C10	0.0 (4)
N1—C2—C3—N1^i^	0.4 (4)	C11—C10—C15—C14	−1.0 (3)
C4—C2—C3—N1^i^	−178.3 (2)	C8—C10—C15—C14	176.6 (2)
N1—C2—C3—C5	−176.6 (2)	C13—C12—O16—C17	15.8 (3)
C4—C2—C3—C5	4.8 (4)	C11—C12—O16—C17	−164.1 (2)
N1^i^—C3—C5—N6	−177.5 (2)	C12—O16—C17—C18	176.2 (2)
C2—C3—C5—N6	−0.4 (4)	O16—C17—C18—C19	−55.3 (3)
N1^i^—C3—C5—O9	−0.4 (3)	C17—C18—C19—C20	−173.5 (2)
C2—C3—C5—O9	176.7 (2)	C18—C19—C20—C21	−168.7 (2)
O9—C5—N6—N7	−0.7 (3)	C19—C20—C21—C22	−179.0 (2)
C3—C5—N6—N7	176.5 (2)	C20—C21—C22—C23	−178.1 (2)
C5—N6—N7—C8	0.2 (3)	C21—C22—C23—C24	179.7 (2)
N6—N7—C8—O9	0.4 (3)	C22—C23—C24—C25	−179.6 (2)
N6—N7—C8—C10	178.7 (2)	C23—C24—C25—C26	−179.2 (2)
N7—C8—O9—C5	−0.8 (3)	C24—C25—C26—C27	−179.9 (2)
C10—C8—O9—C5	−179.2 (2)	C25—C26—C27—C28	−179.9 (2)
N6—C5—O9—C8	0.9 (3)	C15—C14—O29—C30	−1.4 (3)
C3—C5—O9—C8	−176.6 (2)	C13—C14—O29—C30	179.3 (2)
N7—C8—C10—C11	−2.0 (4)	C14—O29—C30—C31	−177.9 (2)
O9—C8—C10—C11	176.2 (2)	O29—C30—C31—C32	178.1 (2)
N7—C8—C10—C15	−179.7 (2)	C30—C31—C32—C33	−166.6 (2)
O9—C8—C10—C15	−1.6 (4)	C31—C32—C33—C34	179.0 (2)
C15—C10—C11—C12	1.1 (4)	C32—C33—C34—C35	−171.7 (2)
C8—C10—C11—C12	−176.6 (2)	C33—C34—C35—C36	179.4 (2)
C10—C11—C12—O16	179.8 (2)	C34—C35—C36—C37	−178.2 (2)
C10—C11—C12—C13	−0.1 (4)	C35—C36—C37—C38	179.8 (2)
O16—C12—C13—C14	179.2 (2)	C36—C37—C38—C39	−179.2 (2)
C11—C12—C13—C14	−0.9 (4)	C37—C38—C39—C40	178.4 (2)
C12—C13—C14—O29	−179.7 (2)	C38—C39—C40—C41	−177.0 (2)
C12—C13—C14—C15	0.9 (4)		

Symmetry code: (i) −*x*+1, −*y*+1, −*z*+1.
